# GBAT: a gene-based association test for robust detection of *trans-*gene regulation

**DOI:** 10.1186/s13059-020-02120-1

**Published:** 2020-08-24

**Authors:** Xuanyao Liu, Joel A. Mefford, Andrew Dahl, Yuan He, Meena Subramaniam, Alexis Battle, Alkes L. Price, Noah Zaitlen

**Affiliations:** 1 Department of Epidemiology, Harvard TH Chan School of Public Health, Boston, MA USA; 2 Department of Human Genetics, The University of Chicago, Chicago, IL USA; 3 Departments of Neurology and Computational Medicine, University of California Los Angeles, Los Angeles, CA USA; 4 Department of Computer Science, Johns Hopkins University, Baltimore, MD USA

**Keywords:** Gene expression, eQTLs, *trans*-eQTLs, *trans* gene regulation

## Abstract

The observation that disease-associated genetic variants typically reside outside of exons has inspired widespread investigation into the genetic basis of transcriptional regulation. While associations between the mRNA abundance of a gene and its proximal SNPs (*cis*-eQTLs) are now readily identified, identification of high-quality distal associations (*trans*-eQTLs) has been limited by a heavy multiple testing burden and the proneness to false-positive signals. To address these issues, we develop GBAT, a powerful gene-based pipeline that allows robust detection of high-quality *trans*-gene regulation signal.

## Introduction

The vast majority of genetic variants associated with complex traits are found in non-coding regions of the genome [1], leading to a natural hypothesis that their effects are mediated through changes in transcriptional regulation. For computational and statistical reasons, efforts to date have focused on mapping *cis-*genetic effects on gene expression despite the fact that *trans*-effects explain more than twice the variability in gene expression than *cis*-effects [2, 3]. Furthermore, while *cis*-genetic effects are widely shared across cell types [4, 5], disease outcomes frequently result from dysregulation of genes in specific cell types [6–10]. In contrast, *trans*-genetic effects are more cell-type-specific [5, 11] and may therefore harbor disease-causing variants not captured in *cis* analyses [12]. It was recently estimated that *trans*-genetic effects to core disease genes could explain 70–100% of the complex trait heritability, and the widespread *trans* effects also underlie the highly polygenic architecture of complex traits [13]. Therefore, detecting and understanding *trans*-genetic effects is a key step towards a complete understanding of complex trait genetics.

However, robust discovery of *trans-*eQTLs is very challenging for several reasons. First, *trans*-effects are typically much smaller than *cis*-effects and thus hard to detect [14]. Second, genome-wide scans for *trans*-eQTLs have heavy burden of multiple testing [3, 14]: a genome-wide *trans*-eQTL test of over twenty thousand genes and one million SNPs results in Bonferroni threshold of 2.5 × 10^−12^. Third, sequencing reads mapping errors, such as multi-mapped reads and reads from repetitive regions, lead to up to 75% false *trans* signals [15, 16]. Fourth, the use of dimensionality reduction techniques to estimate confounding effects, such as PEER [17] or SVA [18], can also be problematic in *trans-*eQTL studies. The use of dimensionality reduction techniques to estimate confounding effects successfully capture confounding factors and improve association power in *cis*-eQTL mapping, but their naïve use in *trans*-eQTL studies can both reduce power [19, 20] and introduce false positives in *trans-*eQTL studies due to collider effects [21]. Indeed, studies introducing PEER, SVA, and other related tools have consistently recommended careful fitting of covariates for detecting *trans*-effects via supervised approaches conditioning on the SNP or the gene of interest. Unfortunately, per-SNP supervision is computationally very expensive in genome-wide *trans*-eQTL scans and is ignored in practice.

Here we address these issues through a new gene-based method for detecting *trans-*effects. Our approach is similar to recent gene-based GWAS approaches (TWAS and PrediXcan) that have proven successful in the context of complex phenotypes [22–24]. Briefly, instead of testing for association between all SNP*-trans* gene pairs, we build cross-validated *cis-*genetic predictions (CVGP) of each gene’s expression and test for association between all CVGP-*trans* gene pairs. This reduces the number of tests by at least two orders of magnitude, substantially improving power to detect *trans*-genetic effects that act through *cis* effects on a gene. Importantly, TWAS and PrediXcan do not work directly for *trans*-eQTL mapping and will produce substantial false positives. We carefully considered all possible sources of power loss and false positives in detecting *trans* signals and made efforts to address these by (1) rigorously filtering out problematic sequencing reads and *trans* signals to reduce false positives due to read mapping errors and (2) properly using supervised versions of dimension reduction approaches. Furthermore, our computational efficient cross-validated *cis*-genetic prediction method does not require external training samples, which is useful when the training samples that match the population background or cell type of study samples are lacking. This also avoided dividing samples into training samples and testing samples, thus maximizes the use of experimental samples.

Through simulations, we showed that our method increased the power of detecting *trans*-gene regulation over other *trans*-eQTL scans, including SNP-based and other gene-based methods [25]. We have implemented our approach for gene-based association test for *trans*-gene regulation in a software pipeline GBAT.

## Results

### GBAT method

**Fig. 1:**
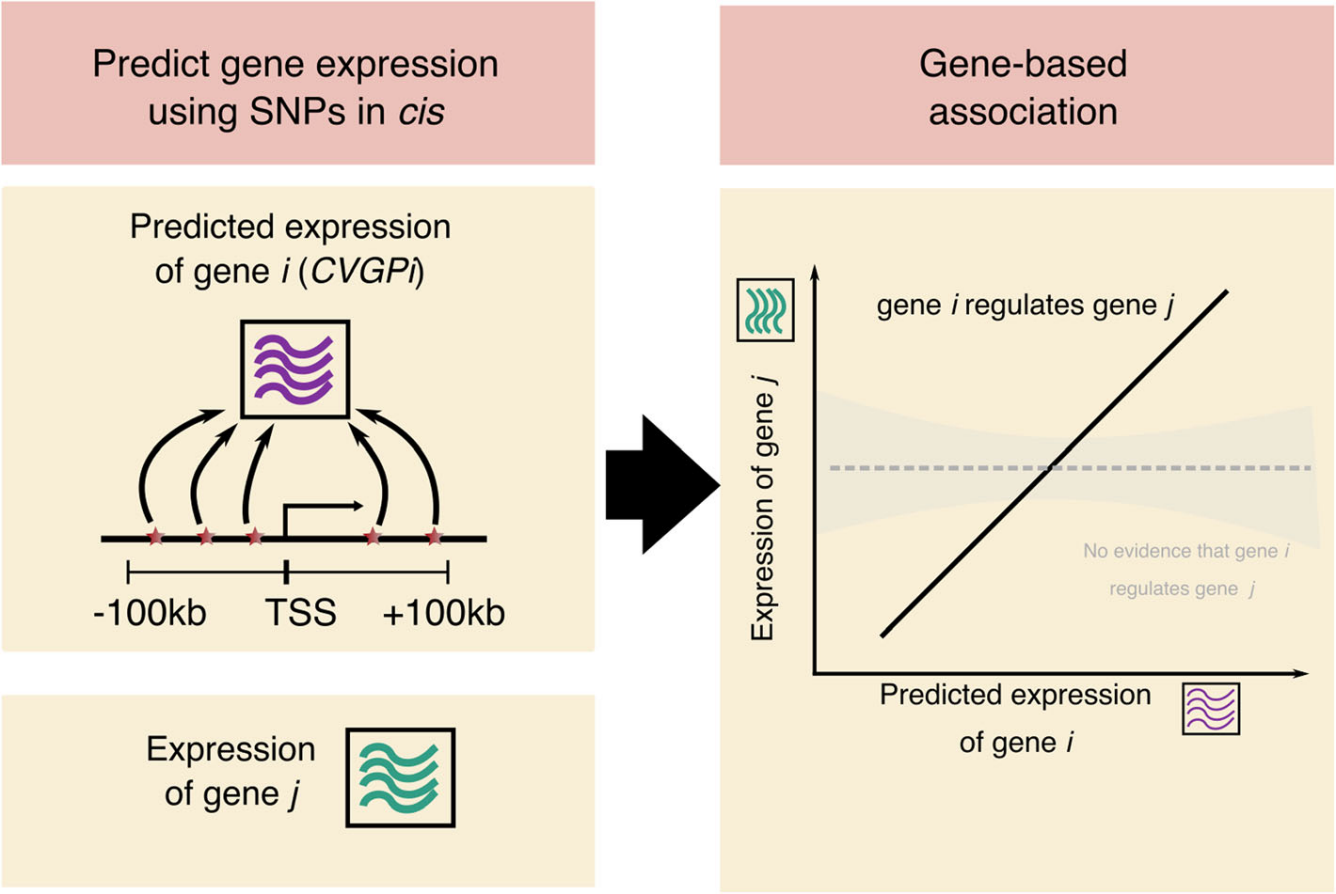
Schematic of the GBAT pipeline. First, GBAT predicts the expression levels from *cis* genetic variants (cross-validated *cis*-genetic prediction of gene *i* (CVGP_*i*_)) using cvBLUP. Then, GBAT performs gene-based association tests between CVGP_*i*_ and expression level of gene *j* to identify gene-based *trans*-association, while properly includes supervised SVA conditional on each CVGP_*i*_ (SV_*i*_) as covariates

GBAT is a gene-based pipeline for detecting high-quality *trans*-gene regulation signals and consists of two main steps (Fig. 1). First, GBAT uses cvBLUP to produce predictions of gene expression from SNPs *cis* to each gene [26]. cvBLUP is a reference-free method that does not rely on external training datasets, but builds leave-one-sample-out cross-validated *cis*-genetic predictions (CVGP_*i*_ for each gene *i*), to avoid overfitting issues of the standard best linear unbiased predictor (BLUP, see the “Methods” section for details). The cvBLUP method dramatically reduces computing time, compared to other leave-one-sample-out cross-validation approaches implemented for prediction methods such as BSLMM [27] and Elastic-net [28]. This gain is attained by building our *N* (*N* = sample size) leave-one-out CVGP predictions after fitting the model only once instead of *N* times [26] (see the “Methods” section for details). Second, we test the association of each CVGP_*i*_ with every *trans* gene *j* (at least 10 Mb away from gene *i*). We first regress the expression levels on covariates to obtain residuals. To make sure the residuals are normal distributed in the regression model, we performed quantile normalization again on the residuals before the association tests.

To test for association, *cis-*eQTL studies typically include covariates such as PEER factors or surrogate variables from SVA that are intended to model confounders [19, 20]. However, PEER factors or SVA could capture large *trans-*effects, and including them as covariates leads to power loss and false positives due to collider effects. To prevent false positives and power loss in *trans-*eQTL studies, the use of supervised versions of PEER and SVA is recommended [21]. For each CVGP_*i*_, we therefore run supervised SVA conditional on CVGP_*i*_, such that the resulting surrogate variable *SV*_*i*_ does not include the genetic effects of gene *i*. We then use *SV*_*i*_ as covariates in the association testing. We note that including conditional SVs as covariate is much more computationally efficient in our gene-based approach than in traditional SNP-based methods for genome-wide scans of *trans*-eQTLs.

RNA sequencing alignment errors can lead to many false-positive *trans* signals [16]. To overcome this problem, we follow ref. [28] to remove multi-mapped reads in the RNA-seq dataset. In addition, to more thoroughly remove problematic reads, we further removed any reads that are mapped to low mappability regions (mappability < 1, see the “Methods” section) of the genome before quantifying gene expression. Finally, we removed any *trans*-gene pairs that are cross-mappable following ref. [16].

### Assessing power of GBAT through simulations

**Fig. 2:**
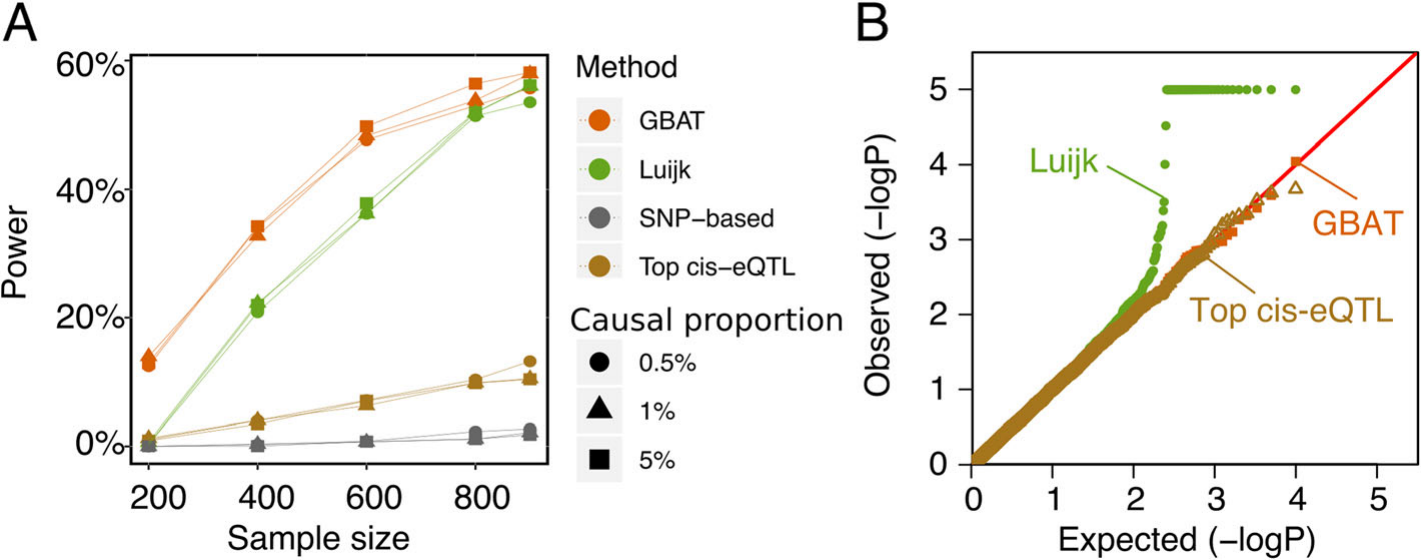
Power and false-positive evaluation of the GBAT approach. **a** Power comparison of GBAT with a gene-based method Luijk et al., the top *cis*-eQTL approach where only the top *cis*-eQTL of a gene is tested for *trans*-eQTL, and the traditional SNP-based *trans*-eQTL scan. The *cis*-heritability was set to 0.1, and the per-gene *trans*-heritability was set to 0.02. Power was assessed at 5% FDR using BH correction and was computed as the fraction of 2000 simulations. Colors represent different methods. **b** Quantile-quantile plot of *trans*-association *p* values from permutation analyses of GBAT, Luijk, and top *cis*-eQTL methods. The *cis*-heritability was set to 0.1, the *trans*-heritability was set to 0.02, sample size is 900, and the causal proportion is at 1%. The simulated expression of the *trans*-gene is randomly permuted for each simulation. The plot is based on 2000 simulations

We performed simulations to assess the power of GBAT for detecting *trans*-effects, in comparison to SNP-based approaches and another gene-based method. Using real genotypes from the RNA sequencing (RNA-seq) dataset of the Depression Genes and Networks study (the DGN dataset, *N* = 913) [15], we simulated gene expression levels, using a causal SNP➜*cis*-expression➜*trans*-expression model with realistic effect sizes (see the “Methods” section). The *cis-*heritability was set to 0.1, and the *trans-*heritability was set to a range of values from 0 to 0.2. We compared the power of GBAT to three alternative *trans*-regulation detection methods: (1) traditional SNP-based *trans*-eQTL scan, (2) SNP-based method that only tests the top *cis*-eQTL of each gene for *trans*-eQTLs, and (3) gene-based method by Luijk et al. in ref. [25]. The Luijk et al. method used one third of the RNA-seq samples as training samples to obtain weights of *cis* regulatory variants through lasso. The weights were then used to predict the genetic values of expression levels in all RNA-seq samples, and *trans*-association was tested between the predicted expression and the expression levels of all *trans* genes. Power was assessed at 5% FDR using Benjamini-Hochberg (BH) and was computed as the fraction of 2000 simulations. Overall, the gene-based approaches (GBAT and Luijk et al.) outperformed SNP-based methods (traditional SNP-based and the top *cis*-eQTL method, Fig. 2a, Additional file 1: Fig. S1A-B), across various simulated genetic architectures of gene expression (proportion of causal SNPs *p* = 0.5%, 1%, and 5%) and different sample sizes (*N* = 200, 400, 600, 800, and 900). GBAT attains better power than the Luijk et al. method at lower sample sizes (*N* = 200, 400, and 600), and GBAT and Luijk et al. have comparable power at higher sample sizes (*N* = 800 and 900). However, we observed higher false-positive rates of Luijk et al. (Additional file 1: Fig. S1C) at higher sample sizes and significant inflation of false positives of the Luijk et al. method in permutation analyses, likely due to overfitting (Fig. 2b). We also evaluated the false-positive inflation of the Luijk et al. method, by only using the remaining two thirds of the samples (instead of all samples) to estimate genetic values of gene expression and test *trans*-associations. However, analogous to Fig. 2b, we observed similar patterns of false-positive inflation in the permutation analyses (Additional file 1: Fig. S1D).

### GBAT produces high-quality *trans*-gene regulation signals in real RNA sequencing dataset

**Fig. 3:**
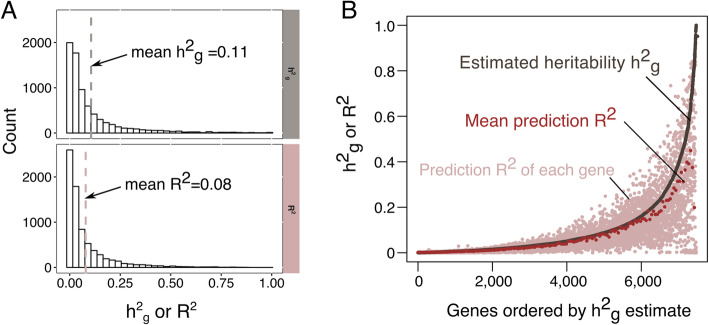
Prediction *R*^*2*^ by cvBLUP in DGN. **a** Histogram of *cis*-${h}_g^2$ (above) and prediction *R*^*2*^ (bottom). **b** Comparison of prediction *R*^*2*^ to *cis*-${h}_g^2$. Gray dots denote *cis*-${h}_g^2$ estimated by REML. Pink dots are the prediction *R*^*2*^ of each gene. Red dots denote the mean prediction *R*^*2*^ for each bin of 50 genes

We next applied GBAT to a whole blood RNA sequencing dataset, the DGN dataset (*N* = 913), to detect *trans-*gene regulation signal. Before quantifying gene expression levels, we first thoroughly remove all reads that are mapped to low mappability regions (mappability< 1.0, see the “Methods” section), in addition to multi-mapped reads. After QC (see the “Methods” section) and removing pseudogenes, expression of 13,447 genes remained. We built cross-validated *cis-*genetic predictions (CVGP) of each gene using cvBLUP with variants within 100 kb of the transcription start site. Prediction accuracy was assessed using squared correlation (prediction *R*^*2*^) between observed and predicted expression levels*.* The average *R*^*2*^ is 0.08 across all genes, and the average *cis* SNP heritability (${h}_g^2$) estimated by restricted maximum likelihood (REML) is 0.11 (Fig. 3a). On average across all genes, the prediction *R*^*2*^ is 85% of the *cis* SNP heritability (${h}_g^2$) (Fig. 3b). We note our prediction accuracy is comparable to prediction methods modeling the sparse genetic architecture of *cis*-gene regulations (Fig. 3 of ref. [22] and Fig. 4 of ref. [23]).

**Fig. 4:**
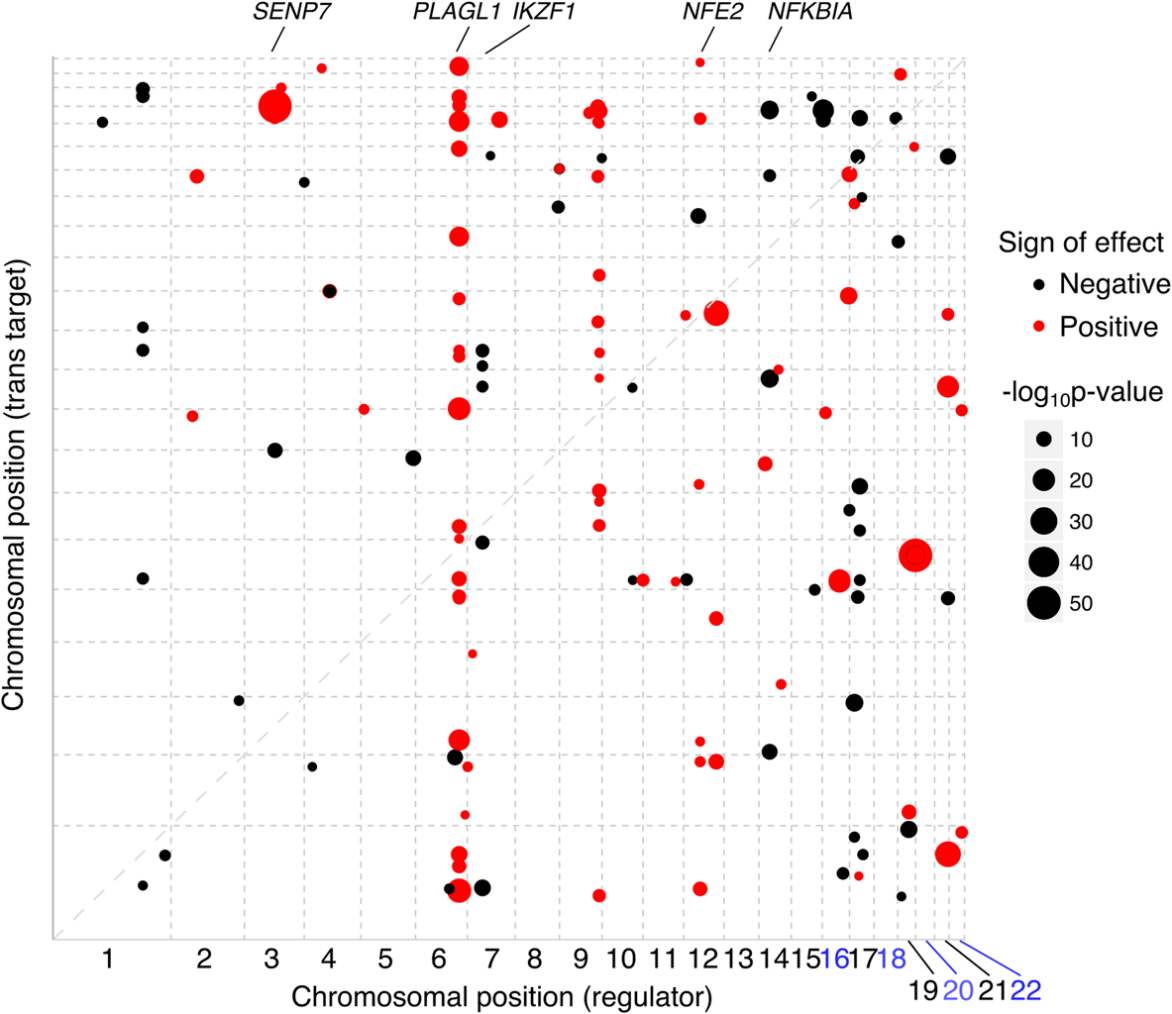
*Trans*-gene regulation signal in DGN. The *X*-axis is chromosomal positions of *trans* regulators, and the *Y*-axis is the *trans* target genes whose expression is regulated by the regulators. The size of the dots denotes the significance of *trans*-association (−log10(*p* value)). The color of the dots denotes the sign of effects. The dashed line is the *y = x* line

We then tested each CVGP with prediction *R*^*2*^ > 0.01 (6535 genes remaining) for association with all genes in *trans* (at least > 10 Mb away, pseudogenes are removed prior to the tests). We computed *q* values ^27^ from the *p* values of all inter-chromosomal gene pairs and applied the threshold to all inter-chromosomal and intra-chromosomal gene pairs. After further removing gene pairs that are cross-mappable due to sequence similarity [15, 16], we identified 166 *trans*-gene pairs, consisting 111 unique regulators (Fig. 4, Additional file 2: Table S1) and 164 *trans*-eGenes. Among the 166 *trans*-gene pairs, 156 (93.9%) are inter-chromosomal (corresponding to 154 unique inter-chromosomal *trans-*eGenes) and 10 (6.1%) are intra-chromosomal (corresponding to 10 unique intra-chromosomal *trans-*eGenes). In contrast, SNP-based eQTL mapping with Matrix eQTL [29] identified only 90 inter-chromosomal *trans*-eGenes at 10% FDR in DGN (see the “Methods” section, Additional file 1: Table S2).

Our GBAT results are highly replicable both within and across studies. Within the DGN dataset, we split the DGN samples into two random non-overlapping subsets to quantify the number of *trans* signals that replicate across the subsets. Treating subset1 (resp. subset2) as the discovery set, 87% (resp. 73%) *trans* signals replicated in the other split, demonstrating high replicability of *trans* signal (Additional file 1: Table S3). We then compared *trans* signals detected by GBAT with the *trans*-eQTL results of Matrix eQTL [29] in DGN. Ninety-two percent of GBAT results are replicated in the Matrix eQTL results at *p* value < 0.05/166. To check replication across different studies, we compared the *trans* signal of DGN to the *trans*-eQTL results of eQTLGen [30], which is the largest whole blood expression dataset to date. One hundred eleven of the 166 (67%) *trans* signal in DGN was also found to be significant at 5% FDR in the eQTLGen. Traditional *trans*-eQTL scans suffer from very low power and high false-positive rates, which result in extremely low replication rates of *trans* signal across different studies [26, 30]. In contrast, GBAT demonstrated high replication rates of *trans* signals across different studies, supporting the ability of GBAT to robustly produce high-quality *trans*-regulation signals.

### *Trans*-regulation signal shed light on *trans*-regulatory mechanisms

To understand the *trans*-gene regulation signals in DGN, we first performed a Gene Ontology enrichment analysis of the *trans* regulators, by using the Database for Annotation, Visualization and Integrated Discovery [31] (DAVID v6.8). We found that the 111 *trans* regulators are highly enriched in categories indicative of transcription factor activity. The top enriched categories include metal ion binding (Benjamini FDR = 7.5 × 10^−6^), nucleic acid binding (Benjamini FDR = 1.5 × 10^−4^), DNA binding (Benjamini FDR = 1.1 × 10^−4^), nucleus (9.3 × 10^−4^), and transcription regulation (Benjamini FDR = 7.6 × 10^−3^, Additional file 1: Table S4). The results support a well-known *trans*-regulatory mechanism that many regulators encode gene products, such as transcription factors, that bind to distant DNA sequences and regulate the expression of the target genes in *trans*.

We identified several known transcription factors that regulate multiple genes in *trans*. For example, *PLAGL1*, a known transcription factor, was detected to regulate expressions of 24 genes in *trans* in the DGN dataset (Fig. 4, Additional file 2: Table S1). *PLAGL1* encodes a zinc finger transcription factor that was previously shown to be a master regulator of the imprinted gene network in mice and human (Iglesias-Platas 2014 Hum Mol Genet, Varrault 2017 Nucleic Acids Res). *NFE2*, which encodes a transcription factor that mainly expresses in hematopoietic cells [32, 33], regulates the expression of six genes in DGN (Fig. 4, Additional file 2: Table S1). The *IKZF1* gene, whose *trans*-regulatory effects have been detected in other *trans*-eQTL studies [14, 25], encodes a transcription factor whose expression is restricted to hemo-lymphopoietic system and is detected to regulate expression of four genes in *trans* in DGN (Fig. 4, Additional file 2: Table S1).

The identification of *trans* regulators that are not transcription factors could elucidate more details of the *trans*-regulation mechanisms. We identified *NFKBIA* (NF-kappa-B inhibitor alpha), a member of NF-kappa-B (NF-kB) inhibitor family, to regulate the expression of three genes in *trans* (Fig. 4, Additional file 2: Table S1). *NFKBIA* itself does not encode a transcription factor. However, it inhibits the activation of the NF-kB family of transcription factors, by sequestering NF-kB in the cytosol and masking their nuclear localization signals [34]. Therefore, instead of directly acting as transcription factors, a possible mechanism of *trans* regulators is to affect the transport of transcription factors in and out of the nucleus.

## Discussion

GBAT significantly improves power and computational efficiency for detecting *trans*-gene regulatory signals using a gene-based approach. GBAT is also carefully designed to reduce false *trans* signal from different sources. First, GBAT reduces false positives caused by RNA-seq alignment errors, by thoroughly removing erroneously mapped RNA-seq reads (multi-mapped reads and reads that are mapped to low mappability regions of the genome) and removing any *trans* gene pairs that are cross-mappable. Similar steps should be taken for all *trans*-regulation analysis, as failure to do so can lead to many false *trans* signals [16]. For example, in the Luijk et al. study, 49 *trans* regulators are reported as significant. However, the study did not correct for RNA-seq read mapping issues, and 15 regulators and the corresponding *trans*-signal are likely false positives: 7 of them have very low mappability (mappability < 0.8), and the remaining 8 are cross-mappable to their *trans* target genes (Additional file 1: Table S5). Second, we used supervised dimension reduction to reduce false *trans* signals due to collider effects [21], and we recommend all gene-based methods to use supervised dimension reduction as it is computationally affordable.

GBAT integrated cvBLUP [26] to efficiently build cross-validated *cis-*genetic expression levels. It allows training and predicting expression levels in the same dataset without overfitting and reduces data harmonization issues when external training dataset with the proper population structure background or cell types are lacking. Very recently, a parallel study of Wheeler et al. applied gene-based approaches PrediXcan and MultiXcan to detect *trans*-regulation signals [35]. The method used external training samples (e.g., GTEx [36]) to obtain the weights of *cis* regulatory SNPs. However, we note that the use of external training samples relies on the assumption that *cis* effects are shared across different datasets or tissues [4, 5]. The *cis* effects that are tissue specific could lead to false inference of *trans*-effects (e.g., a wrong sign of effect) or loss of power (Additional file 1: Fig. S2). Our GBAT pipeline can be easily modified to incorporate gene expression prediction obtained from the prediction method in ref. [35]. However, we suggest following the rest of the GBAT pipeline, which includes vigorous removal of problematic RNA-seq reads before quantifying gene expression, checking mappability and cross-mappability of the *trans* signals, and proper inclusion of supervised surrogate variables in *trans*-association testing.

Our pipeline has several limitations. First, we used the infinitesimal model of cvBLUP to predict gene expression, but the genetic architecture of *cis* gene regulation was shown to be sparse. However, our analysis in DGN demonstrated cvBLUP achieved comparable accuracy in comparison to models incorporating sparsity (Fig. 3). Sparse models of cvBLUP (which are now available) may further improve power. Second, *trans*-regulatory effects that do not function through *cis* expression regulation will not be detected by GBAT. The effects of *trans*-eQTLs might be mediated by other *cis*-regulatory mechanisms such as splicing and methylation, or even mediated by other *trans* mechanisms. Further improvement of GBAT that incorporate these effects will further improve power of *trans*-detection. Third, we note that gene-based associations do not infer causality in either disease or *trans*-gene regulation settings. Genes that colocalize or share *cis* regulatory effects can be detected as *trans* regulators. In our results, we observed several neighboring genes to regulate the same *trans* targets (Additional file 2: Table S1). It is likely that these *trans* signals are tagging the same *trans* regulatory effect, and the inferred regulators are not the causal *trans* regulators. We consolidated the *trans* signals from neighboring genes into one *trans* signal. Further analysis, such as the conditional analyses proposed in ref. [25] can be used to distinguish the tagging genes from the actual regulator. Lastly, mapping issues are not perfectly solved, as mappability and cross-mappability scores do not fully capture sequence similarity across the genome. Sequence similarity and read mapping errors could still lead to artificial *trans* signals.

Given the limitations, we should interpret *trans* signals with extra caution. For example, we found a regulator, *SENP7*, regulates 16 target genes in *trans* in DGN (Additional file 2: Table S1, Fig. 4). The *trans* signal at *SENP7* is also among the top *trans* signal identified by Luijk et al. [25]. Most of the *trans* targets from both studies are genes encoding zinc finger proteins, which are all located on a 250-kb region of chromosome 19. While *SENP7* has zero cross-mappable scores to the target zinc finger genes in DGN (Additional file 1: Table S6), it is highly cross-mappable to several zinc finger genes identified by Luijk et al. (Additional file 1: Table S5). Since zinc finger genes contain repeats and share sequence similarity, it is possible that *SENP7* shares sequence similarity with the *trans* targets in DGN too. Therefore, though cross-mappability scores do not capture the similarity, the *trans* signals could be false signals due to read mapping errors. We also found *SENP7* was previously identified as a *trans*-methylation-QTL locus in blood cell types [37], and *trans*-methylation CpG sites are also in the same 250-kb region on chromosome 19 (Additional file 1: Table S6). The *trans*-meQTLs in *SENP7* were found to have a negative effect on CpG methylation [37], which tends to repress gene expression levels. In our study, we detected *SENP7* to have positive effects on the expression of target genes (Additional file 1: Table S6), which agrees with the negative methylation effects. While the evidence may suggest a *trans*-regulatory mechanism through DNA methylation, we caution that DNA methylation measured by microarray could also be affected by genomic sequence similarity, which leads to false *trans*-methylation signals. Indeed, several *trans*-methylation targets are mapped to genes that are highly cross-mappable to *SENP7* (Additional file 1: Table S6). Though the *trans* signals of *SENP7* are currently included in the results, interpreting the *trans*-regulation signal of *SENP7* requires further investigation (which is out of the scope of this study).

## Methods

### GBAT pipeline

First, as homologous genomic regions with low mappability (such as repeat regions) could lead to bias in RNA-seq read mapping, and significantly increase false *trans*-signals [16], we first thoroughly remove all reads that are mapped to low mappability regions, in addition to multi-mapped reads before quantifying gene expression levels. In more detail, we downloaded the mappability of 36 k-mer of the reference human genome computed by the ENCODE project (see the “Availability of data and materials” section). We define genomic regions with a mappability score < 1 (i.e., 36 k-mers that could be mapped to two or more different genomic regions) as low mappability regions.

Second, GBAT uses cvBLUP [26], a reference-free prediction method that does not rely on external cohort, to produce predictions of gene expression from SNPs *cis* to each gene. The cross-validated prediction by cvBLUP is a cross-validated version of a standard linear mixed model (LMM) prediction, or best linear unbiased predictor (BLUP). We consider an LMM as below:

1 $$ y= X\beta + Zb+\epsilon, \kern0.75em $$

where *y* is the phenotype, in particular the expression of gene, measured on *N* individuals. *X* is a matrix of covariates, including an intercept. *Z* is a standardized *N* × *M* matrix of *M* SNPs within the *cis* region of the gene. *b* is the vector of effect sizes for the SNPs in *Z*, modeled as normally distributed by $\mathbf{b}\sim \mathbf{N}\left(\mathbf{0},\frac{\sigma_{\mathbf{g}}^{\mathbf{2}}}{\mathbf{M}}{\mathbf{I}}_{\mathbf{N}}\right)$. The total *cis*-genetic contribution to the phenotype is then the product *Zb*, with distribution $Zb\sim N\left(\mathbf{0},{\sigma}_g^2K\right)$, where *K* is the genetic relationship matrix defined as $K=\frac{Z{Z}^T}{M}$. Finally,*ϵ* is a vector of non-genetic effects, modeled as $\epsilon \sim \mathbf{N}\left(\mathbf{0},{\sigma}_{\epsilon}^{\mathbf{2}}{\mathbf{I}}_{\mathbf{N}}\right)$. Phenotype *y* therefore has the distribution: *y*~*N*(*Xβ*, ***V***), with $V={\sigma}_g^2K+{\sigma}_{\epsilon}^2{I}_N$. We use standard REML to get estimates of the LMM variance components, ${\hat{\sigma}}_g^2$ and ${\hat{\sigma}}_{\epsilon}^2$. The estimate of the narrow sense heritability ${h}_g^2$ is then the ratio of estimated genetic variance to total variance: ${\hat{h}}_g^2=\frac{{\hat{\sigma}}_g^2}{{\hat{\sigma}}_g^2+{\hat{\sigma}}_{\epsilon}^2}$.

The BLUPs for the random effects are $\hat{b}=\frac{{\hat{\sigma}}_g^2}{M}{Z}^T{\hat{V}}^{-1}\left(y-X\hat{\beta}\right)$, and the genetic predictor or fitted value of *y* is calculated as ${y}_{\mathrm{BLUP}}=Z\hat{b}=Z\frac{{\hat{\sigma}}_g^2}{M}{Z}^T{\hat{V}}^{-1}\left(y-X\hat{\beta}\right)={\hat{\sigma}}_g^2K{\hat{V}}^{-1}\left(y-X\hat{\beta}\right)={\hat{\sigma}}_g^2K{\hat{V}}^{-1}\overset{\sim }{y}$, where $\overset{\sim }{y}=\left(y-X\hat{\beta}\right)$ are the phenotypic residuals after removing the contributions of the covariates *X*.

The standard BLUPs, *y*_BLUP_, overfit the training data, meaning that they are highly correlated with the noise term *ϵ*. Cross-validation is often used to mitigate overfitting. In our analysis, we use a leave-one-out cross-validation scheme to generate a set of out-of-sample LMM predictions: each subject is left out of the dataset in turn; the remaining subjects are used to estimate $\hat{b}$ and then the genetic contribution to the left-out subject’s phenotype is defined using $\hat{b}$.

The resulting collection of cross-validated LMM estimates, or cvBLUPs, is still a strong estimator of the true *cis*-genetic contribution to the phenotype, but does not have spurious correlations with *ϵ*. Fortunately, leave-one-out cross-validation is mathematically simple for BLUPs. Given ${y}_{\mathrm{BLUP}}={\hat{\sigma}}_g^2K{\hat{V}}^{-1}\overset{\sim }{y}=H\overset{\sim }{y}$, such that the prediction is a linear operator $H={\hat{\sigma}}_g^2K{\hat{V}}^{-1}$ applied to $\overset{\sim }{y}$, the out-of-sample prediction and prediction errors can be simply calculated from a single model fit. The cvBLUPs can therefore be calculated in linear time as:

2 $$ {y}_{k, cv\mathrm{BLUP}}=\frac{y_{k,\mathrm{BLUP}}-{H}_{kk}\overset{\sim }{y_i}}{1-{H}_{kk}}, $$

where *y*_*k*, *cv*BLUP_ is the cvBLUP prediction for the left-out sample *k*.

For any gene *i*, the cross-validated *cis*-genetic prediction value (CVGP_*i*_) is calculated with all *cis* genetic variants (± 100 kb to transcription starting sites) of gene *i* by using Eq. (2). The cvBLUP method dramatically reduces computing time, compared to other leave-one-sample-out cross-validation approaches implemented for prediction methods such as BSLMM [27] and Elastic-net [28].

For each CVGP_*i*_ passing a certain prediction *R*^2^ threshold (which depends on the sample size), we test its *trans*-association with expression of all genes > 10 Mb away or on different chromosomes. We first fit the expression of distal genes with covariates, including the supervised surrogate variables conditional on CVGP_*i*_. We then quantile normalize the residuals and test the association between the residuals and CVGP_*i*_.

### Simulations to assess the power of GBAT

We performed simulations to assess the power of gene-based approach (GBAT) for detecting *trans*-effects, in comparison to SNP-based approach. We used real imputed genotypes from DGN (sample size *N* = 913) and simulated a causal SNP➜*cis*-expression➜*trans*-expression model with realistic effect sizes. To simulate the causal relationship of SNP➜*cis*-expression, we randomly chose a gene on chromosome 2 to 22 and simulated the *cis*-expression by using a mixed linear model: *E*_*cis*_ = *Xβ* + *ε*, such that $\beta \sim N\left(0,\frac{\sigma_{cis}^2}{Mp}\right)$ for *p* and *β* = 0 for 1-*p*. Here *M* is the total number of SNPs in the *cis* region of the gene, and *p* is the proportion of causal SNPs. The *cis*-heritability (${\sigma}_{cis}^2\Big)$ was set to 0.1 (the average *cis*-heritability in DGN is 0.11). To match the heavy-tail distribution of gene expression quantified from RNA-seq data, we simulated *ε* to follow a *t*-distribution with 3 degrees of freedoms (variance was scaled to 0.9). We then randomly chose a *trans* gene on chromosome 1 and simulated the causal relationship of *cis-*expression (*E*_*cis*_)➜*trans*-expression (*E*_*trans*_) with a range of *trans* effects, measured by the percentage of variance of *E*_*trans*_ explained by *E*_*cis*_ (i.e. the *trans*-heritability, ranging from 0 to 0.2). To better reflect the imperfect genotyping of the individuals, we assumed that only 10% of the SNPs are genotyped, such that not all causal SNPs are observed. We used all “genotyped” SNPs to estimate predict gene expressions in GBAT and the Luijk et al. method. We also simulated different genetic architectures (proportion of causal SNPs *p* = 0.05%, 1%, and 5%) and sample sizes (*N* = 200, 400, 600, 800, and 900).

We compared the power of GBAT with three other methods: the Luijk et al. method [25], the top *cis*-eQTL approach, and the traditional SNP-based eQTL scans. Power was assessed at 5% FDR using BH for all four methods. For the traditional SNP-based *trans*-eQTL scan, we computed the gene-based FDR by taking the most extreme *p* value per gene, multiplying that *p* value by 1,000,000, and then used BH on the adjusted extreme *p* values, following ref. [36]. False-positive rates were assessed by setting *trans*-effect to be 0 in all simulations. To assess false-positive inflation, we permuted the simulated expression levels of *trans* genes in each simulation.

### Genotype and RNA-seq QC

Genotypes of DGN samples were genotyped on the Illumina HumanOmni 1-Quad BeadChip [15]. Nine hundred twenty-two samples have RNA-seq data available. We further removed related individuals and were left with 913 individuals. We imputed the genotypes on the Michigan Imputation Server [38]. We kept only SNPs with genotyping rate > 99%, minor allele frequency > 5%, and Hardy-Weinberg equilibrium < 10^−6^ using PLINK 2.0 [39].

RNA-seq reads were mapped to the reference genome (NCBI v37) by TopHat in ref. [15]. We further discarded reads that are mapped to multiple locations and reads with > 2 mismatches. Next, we removed genomic regions with low mappability. We quantified expression with HTseq [40]. Expression levels of 13,634 genes with at least 1CPM in at least 50% of the individuals were quantified. Finally, expression levels of these genes are quantified as Transcripts Per Million (TPM). We first quantile normalized the expression levels across samples. Then, we quantile normalized the expression levels to standard normal across genes before running testing for *trans* signals.

### SNP-based *trans*-eQTL mapping in DGN using Matrix eQTL

Matrix eQTL [29] was used to test association between all 13,447 genes used in gene-based testing (after removing pseudogenes) and all imputed variants (MAF > 5%) on different chromosomes of the tested gene with an additive linear model. We included all biological and technical covariates (including expression PCs, genetic PCs, cell type proportions, etc.) available from ref. [15]. The correlation between variant and gene expression levels was evaluated using the estimated *t* statistic from this model. We computed gene-level empirical FDR by first getting the most extreme *p* value per gene. Then, we permuted the sample labels of the expression levels, and further permuted gene labels within each sample. We repeated the association tests for each permutated expression level and inter-chromosomal variants. We took the most extreme *p* value of each gene after the permutation and used it as the empirical NULL *p* values to compute empirical gene-level FDR. We set an FDR threshold of 10%.

### Availability of data and materials

The code for GBAT is available at GitHub (https://github.com/xuanyao/GBAT) [41] and Zenodo (https://zenodo.org/record/3924220, DOI: 10.5281/zenodo.3924220) [42]. The gene expression data and genotype of DGN were downloaded by application through the NIMH Center for Collaborative Genomic Studies on Mental Disorders, under the “Depression Genes and Networks study (D. Levinson, PI)” [15]. The code for simulation is also available at GitHub (https://github.com/xuanyao/GBAT). The ENCODE 36 k-mer of the reference human genome is available for download at http://hgdownload.cse.ucsc.edu/goldenPath/hg19/encodeDCC/wgEncodeMapability/wgEncodeCrgMapabilityAlign36mer.bigWig. We used Matrix eQTL for SNP-based *trans*-eQTL calling, and the software is available at http://www.bios.unc.edu/research/genomic_software/Matrix_eQTL/ [29].

## Supplementary information

**Additional file 1: Supplementary Tables and Figures. Tables S2.** 90 *trans*-eGenes identified by SNP-based trans-eQTL mapping at 10% FDR. **Table S3.** Replication of *trans* signals in subsets of DGN. **Table S4.** Gene-ontology enrichment of 157 regulators identified in DGN at Benjamini-Hochberg (BH) FDR < 0.05. **Table S5.** Significant *trans* regulators in Luijk et al. with low mappability or cross-mappable to targets. **Table S6.** *Trans*-effects of *SENP7* on expression and methylation. **Fig. S1.** Power and false positive rate of GBAT approach in simulations. **Fig. S2.** Consequences of non-shared cis effect in external training data on detecting trans signals in the testing dataset.

**Additional file 2: Supplementary Table S1.** 166 trans signal in DGN detected by GBAT at 10% FDR.

**Additional file 3.** Review history
