# Supplementary material for: GBAT: a gene-based association test for robust detection of trans-gene regulation
Source: Genome Biol. 2020 Aug 24;21:211. doi: 10.1186/s13059-020-02120-1 (PMC7444084; doi:10.1186/s13059-020-02120-1)
Supplement: Supplementary file 1 — Additional file 1: Supplementary Tables and Figures. Tables S2. 90 trans-eGenes identified by SNP-based trans-eQTL mapping at 10% FDR. Table S3. Replication of trans signals in subsets of DGN. Table S4. Gene-ontology enrichment of 157 regulators identified in DGN at Benjamini-Hochberg (BH) FDR < 0.05. Table S5. Significant trans regulators in Luijk et al. with low mappability or cross-mappable to targets. Table S6. Trans-effects of SENP7 on expression and methylation. Fig. S1. Power and false positive rate of GBAT approach in simulations. Fig. S2. Consequences of non-shared cis effect in external training data on detecting trans signals in the testing dataset. [file 13059_2020_2120_MOESM1_ESM.docx]

**Supplementary Tables**

**Table S2. 90 *trans-*eGenes identified by SNP-based *trans-*eQTL mapping at 10% FDR.** *Trans-*eQTL mapping were performed with Matrix eQTL. Gene-level empirical FDR were obtained by using empirical p values from permutations (See supplementary notes). The P.value column is the extreme p value of each gene reported by Matrix eQTL. Genes are ordered by P.values.

| **Gene** | **P.value** | **Gene** | **P.value** | **Gene** | **P.value** |
| --- | --- | --- | --- | --- | --- |
| LOC399744 | 1.55E-91 | ZNF547 | 1.61E-21 | ESAM | 4.92E-17 |
| ZNF418 | 2.89E-55 | GUCY1A3 | 9.51E-21 | ZBTB47 | 6.86E-17 |
| ZNF154 | 9.79E-38 | HCP5 | 1.61E-20 | SLC6A4 | 1.09E-16 |
| FLJ45445 | 2.85E-37 | U4 | 2.06E-20 | ZIK1 | 1.13E-16 |
| TTC9 | 1.03E-35 | TSPAN9 | 6.13E-20 | RNF144A-AS1 | 1.43E-16 |
| DUSP22 | 3.37E-35 | ZNF835 | 8.24E-20 | CMTM5 | 2.55E-16 |
| LOC440311 | 2.09E-32 | HOMER2 | 1.12E-19 | ANKRD22 | 3.34E-16 |
| JAM3 | 5.04E-32 | LOC646214 | 1.72E-19 | SEC14L5 | 3.71E-16 |
| BTN3A1 | 3.38E-31 | MAPKAPK3 | 1.91E-19 | MYL4 | 4.23E-16 |
| PARVB | 7.37E-30 | GUCY1B3 | 1.99E-19 | ITGA2B | 4.47E-16 |
| ZSCAN18 | 3.50E-29 | PKHD1L1 | 2.08E-19 | ZNF687 | 6.10E-16 |
| DSE | 3.10E-27 | SLC24A3 | 2.57E-19 | ZNF329 | 7.85E-16 |
| ZNF671 | 1.03E-26 | TTC7B | 3.33E-19 | PF4 | 9.34E-16 |
| PCSK6 | 1.53E-26 | LTBP1 | 4.02E-19 | LOC729176 | 9.41E-16 |
| MMRN1 | 4.37E-25 | VSIG2 | 5.27E-19 | HAUS5 | 1.21E-15 |
| PAQR7 | 2.56E-24 | SELP | 5.57E-19 | ADCY6 | 1.25E-15 |
| HGD | 8.07E-24 | ZNF577 | 2.23E-18 | TGM3 | 2.41E-15 |
| ITGB3 | 9.83E-24 | LY6G6F | 2.75E-18 | MYL9 | 2.99E-15 |
| ZNF211 | 1.01E-23 | ILK | 6.12E-18 | EGR3 | 4.00E-15 |
| ZNF304 | 1.63E-23 | ZNF274 | 6.43E-18 | RAB27B | 4.04E-15 |
| ABLIM3 | 2.28E-23 | SH3BGRL2 | 1.53E-17 | ZNF419 | 5.01E-15 |
| ZNF135 | 3.75E-23 | C6orf25 | 1.82E-17 | LOC200772 | 6.23E-15 |
| POP1 | 4.36E-23 | ACSBG1 | 1.88E-17 | HCG26 | 8.55E-15 |
| ALOX12 | 9.62E-23 | VAV1 | 1.89E-17 | HKR1 | 8.91E-15 |
| PPBP | 2.66E-22 | PTGS1 | 2.15E-17 | C15orf26 | 1.00E-14 |
| PGLS | 4.22E-22 | TCRBV5S1A1T | 2.66E-17 | TUBA8 | 1.06E-14 |
| KIAA0319L | 5.88E-22 | NRGN | 3.10E-17 | PRKAR2B | 1.28E-14 |
| SAMD14 | 8.16E-22 | ZNF595 | 3.17E-17 | CLU | 1.50E-14 |
| GP1BA | 8.32E-22 | LOC100288778 | 3.43E-17 | KCNJ15 | 2.31E-14 |
| GP6 | 1.10E-21 |  |  | FERMT3 | 2.98E-14 |
| ZNF551 | 1.31E-21 |  |  |  |  |

**Table S3. Replication of trans signals in subsets of DGN.** (A) Replication of subset1 *trans-* signals in subset2. (B) Replication of subset2 *trans-* signals in subset1

(A) Replication of subset1 *trans-* signals in subset2.

| **Regulator** | **Target** | **replication.pvalue.subset2** |
| --- | --- | --- |
| ZNF497 | DSE | 8.37E-17 |
| PRDM4 | AVIL | 1.90E-11 |
| PLAGL1 | PAQR7 | 1.44E-10 |
| NLRC5 | BTN3A1 | 2.43E-09 |
| PKNOX1 | ZNF687 | 8.58E-09 |
| CBS | ZNF687 | 2.01E-08 |
| NLRC5 | HCP5 | 2.33E-08 |
| CBS | DUSP13 | 2.01E-07 |
| PKNOX1 | DUSP13 | 4.08E-07 |
| ZFP82 | NID1 | 3.70E-06 |
| CETP | BTN3A1 | 4.74E-06 |
| PEX12 | FKBP8 | 1.35E-05 |
| PEX12 | DMTN | 0.00068824 |
| A1BG | MACROD1 | 0.49131254 |
| INSC | REPIN1 | 0.77820712 |

(B) Replication of subset2 *trans-* signals in subset1

| **Regulator** | **Target** | **replication.pvalue.subset1** |
| --- | --- | --- |
| PKNOX1 | ZNF687 | 2.00E-13 |
| CBS | ZNF687 | 3.23E-12 |
| ZNF497 | DSE | 4.78E-12 |
| NLRC5 | BTN3A1 | 1.39E-11 |
| PRDM4 | AVIL | 1.92E-11 |
| NLRC5 | HCP5 | 4.63E-09 |
| PLAGL1 | PAQR7 | 3.27E-08 |
| PWP1 | AVIL | 1.08E-07 |
| ZNF75A | ZNF230 | 4.61E-06 |
| PLAGL1 | MAPKAPK3 | 0.0001986 |
| PIGL | TBL1XR1 | 0.00032985 |
| CENPV | TBL1XR1 | 0.00741691 |
| TTC19 | TBL1XR1 | 0.00944405 |
| ADORA2B | TBL1XR1 | 0.0511463 |
| ZNF829 | ATP6V1E2 | 0.09278491 |

**Table S4. Gene-ontology enrichment of 157 regulators identified in DGN at Benjamini-Hochberg (BH) FDR<0.05.** Analysis was performed by using DAVID 6.8(Huang et al. 2009). Table is ordered by BH FDR.

| **Category** | **Term** | **Count** | **Genes** | **Fold Enrichment** | **Benjamini** |
| --- | --- | --- | --- | --- | --- |
| GOTERM_MF_DIRECT | GO:0046872~metal ion binding | 31 | ZNF276, HKR1, ZNF517, ZNF132, ZNF75A, ZNF34, ACAT1, ZNF251, PLAGL1, ZSCAN22, ZNF709, MCEE, ZFP90, U2AF1, NSF, ZNF266, MAP2K5, ZNF641, IKZF1, ZFP30, ZBTB40, ZNF497, ZNF585A, PMM1, ZBTB25, ZFP82, PRDM4, MZF1, NEK8, PPP2R3C, CBS | 2.90723488 | 7.49E-06 |
| GOTERM_MF_DIRECT | GO:0003676~nucleic acid binding | 19 | ZNF276, ZNF641, HKR1, ZNF517, ZNF132, IKZF1, ZFP30, CPEB4, ZBTB40, ZNF75A, ZNF34, ZNF585A, ZNF251, ZFP82, ZNF709, ZFP90, CLEC2D, MZF1, ZNF266 | 3.74279713 | 1.51E-04 |
| GOTERM_MF_DIRECT | GO:0003677~DNA binding | 25 | ZNF276, ZNF641, HKR1, ZNF517, HIST1H2BD, ZNF132, IKZF1, ZFP30, ZBTB40, ZNF75A, ZNF497, ZNF34, AHR, ZNF251, ZBTB25, PLAGL1, HHEX, ZFP82, PKNOX1, PRDM4, ZNF709, ZFP90, PSMC3IP, PARP2, ZNF266 | 2.89776707 | 1.08E-04 |
| GOTERM_CC_DIRECT | GO:0005634~nucleus | 47 | ZNF276, HKR1, ZNF517, ZNF132, FIGNL1, CPEB4, NFKBIA, ZNF75A, ZNF34, SENP7, ZNF251, ATF1, PLAGL1, NLRC5, ZSCAN22, PKN3, ZNF709, ZFP90, PSMC3IP, TEF, QKI, GAPDH, ZNF266, MAP2K5, ZNF641, NFE2, HIST1H2BD, IKZF1, CSNK1G2, SMG5, ZFP30, ZBTB40, RPS9, ZNF497, UBN2, ZNF585A, AHR, PWP1, ZFP82, HHEX, PKNOX1, PRDM4, MZF1, PARP2, PPP2R3C, NEK6, CBS | 1.77726872 | 9.29E-04 |
| GOTERM_BP_DIRECT | GO:0006355~regulation of transcription, DNA-templated | 22 | ZNF276, HKR1, ZNF517, ZNF132, ZFP30, ZBTB40, ZNF75A, ZNF497, ZNF34, ZNF585A, AHR, ZNF251, ZBTB25, ZFP82, PKNOX1, ZSCAN22, PRDM4, ZNF709, ZFP90, MZF1, EAF2, ZNF266 | 2.82330643 | 0.00762557 |
| GOTERM_BP_DIRECT | GO:0006351~transcription, DNA-templated | 25 | ZNF276, ZNF641, HKR1, ZNF517, NFE2, ZNF132, IKZF1, ZFP30, ZBTB40, ZNF75A, ZNF497, ZNF34, ZNF585A, AHR, ZNF251, PWP1, ZBTB25, PLAGL1, HHEX, ZFP82, ZSCAN22, ZNF709, ZFP90, MZF1, ZNF266 | 2.46817768 | 0.00701688 |
| GOTERM_MF_DIRECT | GO:0003700~transcription factor activity, sequence-specific DNA binding | 15 | ZNF641, NFE2, ZNF132, IKZF1, ZFP30, ZNF585A, AHR, ATF1, ZNF251, ZBTB25, PLAGL1, ZFP82, PKNOX1, ZSCAN22, MZF1 | 3.02863397 | 0.0136026 |

**Table S5. Significant *trans* regulators in Luijk et al with low mappability or cross-mappable to targets.**

| Gene.name..index. | Gene.name..target. | Chr..index. | Chr..target. | Cross.map R-T | Cross.map T-R | Mappability of Regulator |
| --- | --- | --- | --- | --- | --- | --- |
| LRRC37A | LRRC37A3 | 17 | 17 | 5007.5 | NA | 0.227 |
| LGALS9C | USP6 | 17 | 17 | NA | NA | 0.430 |
| RABL2B | RABL2A | 22 | 2 | 4403.5 | NA | 0.474 |
| SOD2 | FAM177B | 6 | 1 | 111.5 | NA | 0.651 |
| SLC2A3 | HGD | 12 | 3 | NA | NA | 0.711 |
| FKBP1A | FKBP1C | 20 | 6 | 1437.5 | NA | 0.730 |
| EIF5A | EIF5AL1 | 17 | 10 | 1002.5 | NA | 0.773 |
| ZNF439 | AIG1 | 19 | 6 | NA | 11 | 0.808 |
| PABPC1 | PABPC3 | 8 | 13 | 727.5 | NA | 0.834 |
| SENP7 | HKR1 | 3 | 19 | 28.5 | NA | 0.952 |
| SENP7 | ZNF329 | 3 | 19 | 50.5 | NA | 0.952 |
| SENP7 | ZIK1 | 3 | 19 | 30.5 | NA | 0.952 |
| SENP7 | ZNF154 | 3 | 19 | 19 | NA | 0.952 |
| SENP7 | ZNF274 | 3 | 19 | 6 | NA | 0.952 |
| SENP7 | ZNF577 | 3 | 19 | 3 | NA | 0.952 |
| SENP7 | ZNF626 | 3 | 19 | 29.5 | NA | 0.952 |
| SENP7 | ZNF419 | 3 | 19 | NA | 2 | 0.952 |
| SENP7 | ZNF486 | 3 | 19 | 12.5 | NA | 0.952 |
| SENP7 | ZNF814 | 3 | 19 | 39 | NA | 0.952 |
| SENP7 | ZNF835 | 3 | 19 | NA | 22 | 0.952 |
| SENP7 | ZNF737 | 3 | 19 | 3.5 | NA | 0.952 |
| SENP7 | ZNF773 | 3 | 19 | 32 | NA | 0.952 |
| SENP7 | ZSCAN18 | 3 | 19 | NA | 1.5 | 0.952 |
| GPS2 | EIF5AL1 | 17 | 10 | 123.5 | NA | 0.959 |
| BAZ2B | KIAA0319L | 2 | 1 | 57 | NA | 0.966 |
| BAZ2B | MANSC1 | 2 | 12 | NA | 15.5 | 0.966 |
| SAMD3 | DGKD | 6 | 2 | NA | 2.5 | 0.978 |
| SAMD3 | FAM153B | 6 | 5 | 13 | NA | 0.978 |
| SAMD3 | FAM153A | 6 | 5 | 13 | NA | 0.978 |
| CREB5 | EXTL3 | 7 | 8 | NA | 1.5 | 0.990 |
| CREB5 | KIAA0319 | 7 | 6 | NA | 4 | 0.990 |
| SP110 | ZNF418 | 2 | 19 | 1 | NA | 1.000 |

**Table S6. *Trans*-effects of *SENP7* on expression and methylation.** (A) Significant *trans* effects of *SENP7* on ZNF genes on chromosome 19 in DGN. (B) Significant effects of *SENP7* on distal CpG sites from Ref (Lemire et al. 2015) to distal CpG sites on chromosome 19. CpG annotated to genes that are cross mappable to *SENP7* are highlighted in red.

(A) Significant *trans* effects of *SENP7* on ZNF genes on chromosome 19 in DGN.

| Regulator | Target | Regulator.pos | Target.chr | Target.start | Target.end | p.value | Effect |
| --- | --- | --- | --- | --- | --- | --- | --- |
| SENP7 | ZNF418 | chr3: 101043118-101232085 | chr19 | 58433252 | 58446740 | 4.96E-50 | 0.46 |
| SENP7 | ZNF671 | chr3: 101043118-101232085 | chr19 | 58231119 | 58238995 | 3.76E-27 | 0.35 |
| SENP7 | ZNF211 | chr3: 101043118-101232085 | chr19 | 58131794 | 58154147 | 2.03E-20 | 0.30 |
| SENP7 | ZNF304 | chr3: 101043118-101232085 | chr19 | 57862645 | 57871265 | 1.26E-19 | 0.29 |
| SENP7 | ZNF135 | chr3: 101043118-101232085 | chr19 | 58570607 | 58581110 | 2.22E-19 | 0.29 |
| SENP7 | ZNF551 | chr3: 101043118-101232085 | chr19 | 58193337 | 58201169 | 7.48E-19 | 0.29 |
| SENP7 | TRAPPC2P1 | chr3: 101043118-101232085 | chr19 | 57874879 | 57876716 | 5.59E-18 | 0.28 |
| SENP7 | ZNF547 | chr3: 101043118-101232085 | chr19 | 57874879 | 57890925 | 1.39E-17 | 0.28 |
| SENP7 | ZNF134 | chr3: 101043118-101232085 | chr19 | 58125830 | 58133636 | 2.95E-15 | 0.26 |
| SENP7 | ZNF256 | chr3: 101043118-101232085 | chr19 | 58452201 | 58459077 | 1.15E-12 | 0.23 |
| SENP7 | ZNF416 | chr3: 101043118-101232085 | chr19 | 58082934 | 58090243 | 1.20E-11 | 0.22 |
| SENP7 | ZNF667-AS1 | chr3: 101043118-101232085 | chr19 | 56989243 | 57006805 | 1.45E-11 | 0.22 |
| SENP7 | ZNF649 | chr3: 101043118-101232085 | chr19 | 52392488 | 52408305 | 2.79E-10 | 0.21 |
| SENP7 | ZNF264 | chr3: 101043118-101232085 | chr19 | 57702868 | 57734214 | 2.47E-07 | 0.17 |

(B) Significant effects of *SENP7* on distal CpG sites from Ref (Lemire et al. 2015) to distal CpG sites on chromosome 19. CpG annotated to genes that are cross mappable to *SENP7* are highlighted in red.

| SNP | Chr | SNPPos | CpG | CpGChr | CpGPos | Gene annotations | CpG location |
| --- | --- | --- | --- | --- | --- | --- | --- |
| rs2141180 | 3 | 101179057 | cg01268824 | 19 | 58220818 | ZNF154 | TSS1500 |
| rs2141180 | 3 | 101179057 | cg01644850 | 19 | 58193231 | ZNF551 | TSS200 |
| rs2141180 | 3 | 101179057 | cg11294513 | 19 | 58220295 | ZNF154 | Body |
| rs2141180 | 3 | 101179057 | cg15746696 | 19 | 58400494 | ZNF814 | TSS200 |
| rs2141180 | 3 | 101179057 | cg27049766 | 19 | 58220516 | ZNF154 | 5'UTR |
| rs2141180 | 3 | 101179057 | cg27112264 | 19 | 58400504 | ZNF814 | TSS200 |
| rs2553419 | 3 | 101068620 | cg22510337 | 19 | 58399967 | ZNF814 | Body |
| rs2682386 | 3 | 101068873 | cg04684267 | 19 | 58694502 | ZNF274/ZNF274 | 1stExon/5'UTR |
| rs2682386 | 3 | 101068873 | cg24214260 | 19 | 58193221 | ZNF551 | TSS200 |
| rs2682386 | 3 | 101068873 | cg26465391 | 19 | 58220773 | ZNF154 | TSS200 |
| rs2682386 | 3 | 101068873 | cg27324426 | 19 | 58220837 | ZNF154 | TSS1500 |
| rs9859077 | 3 | 101136402 | cg03234186 | 19 | 58220657 | ZNF154 | TSS200 |
| rs9859077 | 3 | 101136402 | cg06458239 | 19 | 58038573 | ZNF549 | TSS200 |
| rs9859077 | 3 | 101136402 | cg06615754 | 19 | 58193071 | ZNF551 | TSS1500 |
| rs9859077 | 3 | 101136402 | cg08668790 | 19 | 58220662 | ZNF154 | TSS200 |
| rs9859077 | 3 | 101136402 | cg10729426 | 19 | 58038585 | ZNF549 | TSS200 |
| rs9859077 | 3 | 101136402 | cg22299467 | 19 | 58399865 | ZNF814 | Body |
| rs9859077 | 3 | 101136402 | cg25400396 | 19 | 58400559 | ZNF814 | TSS200 |

**Supplementary Figures**

**Figure S1. Power and false positive rate of GBAT approach in simulations.** We simulated realistic *cis* and *trans* effects. The cis-heritability was set to 0.1 and the trans-heritability was set to values range from 0-0.2. Power was computed as the fraction of 2000 simulations where significant association was identified. Colors of points represent different methods. (A-B). Power comparison at trans h^2^g=0.2 and 0.002. (C) False positive rates of *trans* methods, when trans h^2^g=0. (D) Quantile-quantile plot of p values from permutation analysis. Trans h^2^g=0.02.


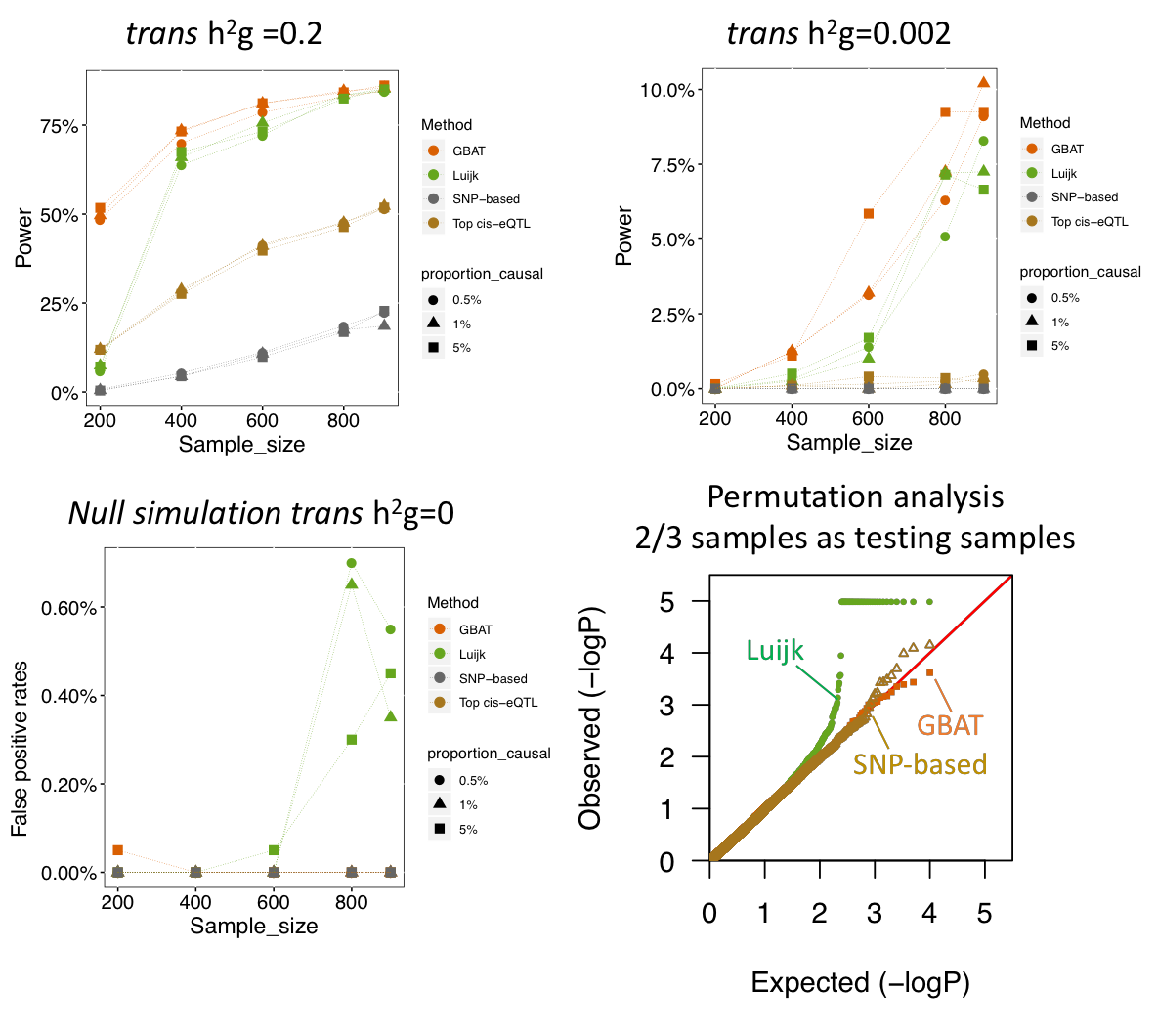


**Figure S2. Consequences of non-shared *cis* effect in external training data on detecting *trans* signals in the testing dataset.** We list two scenarios under which non-shared cis effects in the training dataset could lead to loss of power or wrong inference of trans effect size.


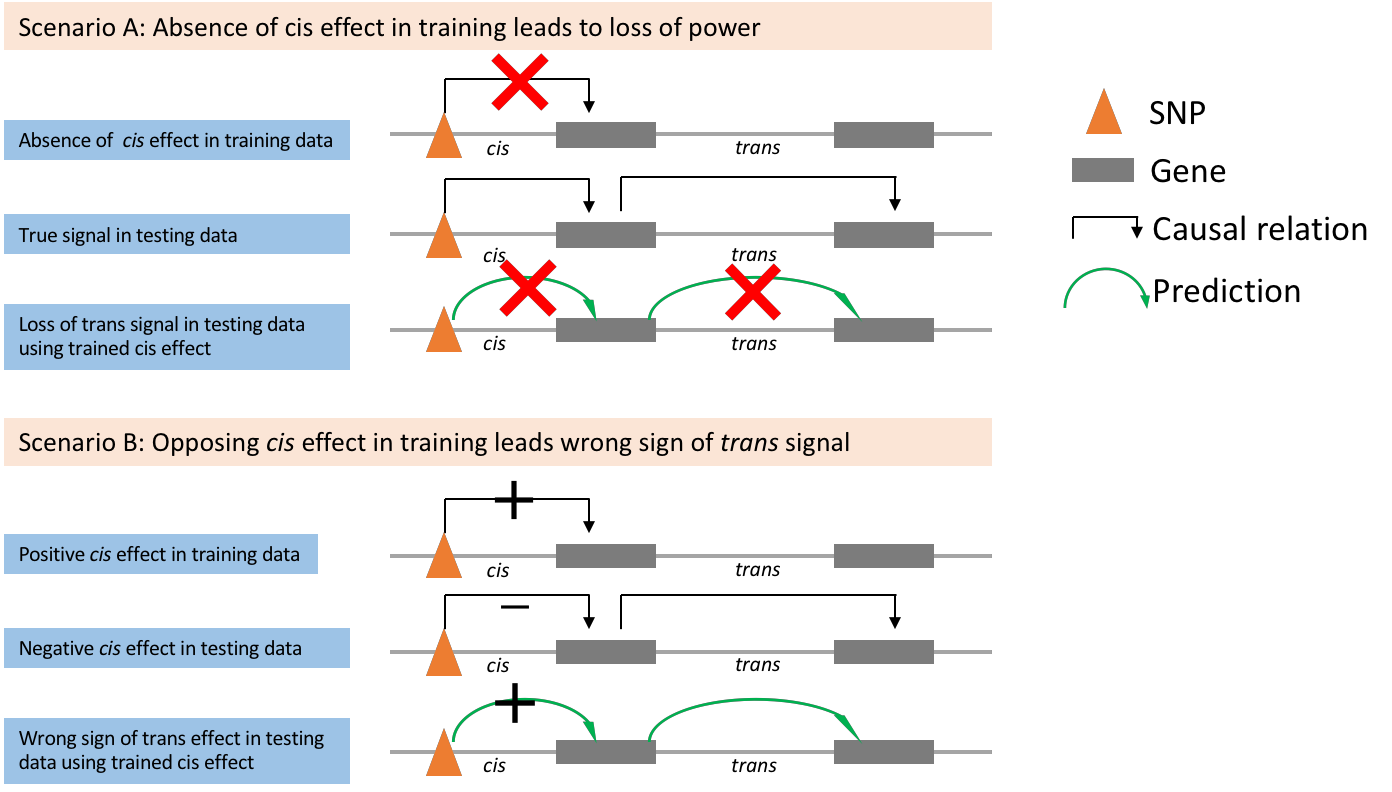


Bibliography

Huang, D.W., Sherman, B.T. and Lempicki, R.A. 2009. Systematic and integrative analysis of large gene lists using DAVID bioinformatics resources. *Nature Protocols* 4(1), pp. 44–57.

Lemire, M., Zaidi, S.H.E., Ban, M., et al. 2015. Long-range epigenetic regulation is conferred by genetic variation located at thousands of independent loci. *Nature Communications* 6, p. 6326.
