# Supplementary material for: GBAT: a gene-based association test for robust detection of trans-gene regulation
Source: Genome Biol. 2020 Aug 24;21:211. doi: 10.1186/s13059-020-02120-1 (PMC7444084; doi:10.1186/s13059-020-02120-1)
Supplement: Supplementary file 3 — Additional file 3. Review history [file 13059_2020_2120_MOESM3_ESM.docx]

**Review history**

**First round of review**

**Reviewer 1**

Liu et al describe a method that can identify trans-eQTL effects, without suffering from the problem that trans-eQTL analyses need to correct for billions of statistical tests. The strategy the authors take is to first identify a set of cis-eQTL SNPs, and to predict cis-gene expression and to subsequently correlate this predicted cis-gene expression to other genes. As such the number of tests goes down over a thoudand fold, and power improves to identify trans-eQTLs, under one major assumption: that SNPs that give trans-eQTL effects always give cis-eQTL effects.

I have one major comment, and that is the fact that this manuscript describes a strategy that is nearly identical to what has been published before in two manuscripts:
- Imputed gene associations identify replicable trans-acting genes enriched in transcription pathways and complex traits: https://onlinelibrary.wiley.com/doi/abs/10.1002/gepi.22205?af=R
- Genome-wide identification of directed gene networks using large-scale population genomics data: https://www.nature.com/articles/s41467-018-05452-6

It is a pity the authors have not been aware of these methods. To further complicate matters, the first paper also uses the same dataset as Liu to show the concept to work. As such I have strong reservations on the novelty of the approach. I leave it up to the editor to decide whether it is worthwhile to still consider this paper for Genome Biology, but honestly do not feel the need to comment on specific aspects of the paper, because this can only be done when taking into consideration the two papers that have been published before.

**Reviewer 2**

Main comments

1.     The concept of using a cis-region based genetic predictor is interesting in that it should capture the summed genetic variance in the region. However, the strategy of reducing the test space by only fitting trans-eQTL between a significant cis-eSNP and other genes has been done routinely. One of the most powerful examples being in Westra et al. Nature Genetics 2013. Where they use the cis-eQTL top eQTL. I can see how the CVGP allows genetic effects of multiple cis-eQTL to be captured, it will be estimated with considerable great error than the top cis-eQTL. In evaluating the performance of GBAT, benchmarking over using the common cis-SNP filtering / test would be very helpful to readers.

2.     In results page 2 lines 45-48 it is noted that including conditional CV as covariates is impossible in SNP based trans-associations. Firstly, it is not clear why this this impossible. Secondly, the common was to include SV is to use them to first correct the expression levels of gene i, then use the corrected values in the eQTL analysis. Some additional clarification and justification of these statements would be beneficial. Regressing normalised Ej on SVi has also been done by others - for example see Lloyd-Jones et al. AJHG 2017.

3.     Demonstration of the performance of GBAT needs to show that replication is higher compared to other methods. Rather than being 'out of scope' I believe this is would provide a critical piece of information for the manuscript and in convincing researchers to adopt the method.

4.     Filtering multi-mapped reads is of course important. However, this is very standard in most RNA seq studies. For example, it is default in HTseq, eQTL-cookbook, and the GTEx pipelines. I think the paper would benefit from not positioning this as a key step. It detracts from the novelty, and also leads to the impression that most other analysis pipelines/tools do not follow this approach, which is not true.

5.     The inflation of the false positive rate using the regression model E_j = CVGP_i + SV_i is intriguing. It is not clear why this is happening given that correcting E_j by SV_i then fitting E_j = CVGP_i is ok. The paper would benefit considerably if an explanation for the inflation can be demonstrated.

6.     The need to reduce the number of tests is important, but the approach of only testing cis-eQTL means that all 'trans-trans' eQTL will be missed. This is worth some discussion as it is anticipated that the majority of genetic variation for gene expression is acting trans-trans, although with smaller effects than cis-trans. The 1 sentence in the discussion just mentioned this as a limitation.

7.     Simulating 10% of SNPs being causal is extremely high and unrealistic. Some justification for these percentages needs to be given based on what is known about eQTLs.

8.     It is unclear if the power of GBAT was tested at the same alpha level to SNP-based methods, or at a test-specific multiple testing corrected thresholds. It should be compared at the same alpha.  

9.     The results reporting the GO enrichment are underwhelming, and it is not clear what conclusions are drawn from these. Statements such as 'the results are encouraging as these categories are expected to have broad trans effects' really should be supported by references or qualified.


Minor comments

1.     Slightly pedantic, but using the word 'suffer' (line 22) when describing multiple testing correct can give the impression that multiple testing is a bad thing. Could this be re-phrased to explain that the issue is one of power? Irrespective of effect sizes, the nominal alpha level that needs to be reached is smaller, thus given the same effect size large sample sizes are needed.
2.     The comment that cis-eQTL are largely shared across tissues is not really true. The GTEX papers showed that some cis-EQTL are truly tissue ubiquitous, many are shared by only a handful of tissue types, and some tissue specific.  
3.     Are some of the methods are missing? The methods section starts with 'Second, GBAT uses…. 'It's possible this is a typo, as most of the key methods descriptions are included.'

**Authors’ response to reviewers**

Reviewer #1: Liu et al describe a method that can identify trans-eQTL effects, without suffering from the problem that trans-eQTL analyses need to correct for billions of statistical tests. The strategy the authors take is to first identify a set of cis-eQTL SNPs, and to predict cis-gene expression and to subsequently correlate this predicted cis-gene expression to other genes. As such the number of tests goes down over a thoudand fold, and power improves to identify trans-eQTLs, under one major assumption: that SNPs that give trans-eQTL effects always give cis-eQTL effects.

I have one major comment, and that is the fact that this manuscript describes a strategy that is nearly identical to what has been published before in two manuscripts:
- Imputed gene associations identify replicable trans-acting genes enriched in transcription pathways and complex traits: https://onlinelibrary.wiley.com/doi/abs/10.1002/gepi.22205?af=R
- Genome-wide identification of directed gene networks using large-scale population genomics data: https://www.nature.com/articles/s41467-018-05452-6

It is a pity the authors have not been aware of these methods. To further complicate matters, the first paper also uses the same dataset as Liu to show the concept to work. As such I have strong reservations on the novelty of the approach. I leave it up to the editor to decide whether it is worthwhile to still consider this paper for Genome Biology, but honestly do not feel the need to comment on specific aspects of the paper, because this can only be done when taking into consideration the two papers that have been published before.

*We thank the reviewer for the comments. We agree with the reviewer that the gene-based approach for detecting trans regulatory signals is not a novel idea proposed by our manuscript. Both methods mentioned by the reviewer (Luijk et al 2018 and Wheeler et al 2019) are also using a similar approach. The Luijk et al method uses lasso to train cis-eQTL weights in one third of the samples, and uses the weights to predict genetic values of gene expression in all samples. The method then tests association between the predicted value and the expression of all genes in trans. The Wheeler et al applied the PrediXscan and MultiXscan methods in trans gene regulation setting. However, we emphasize that our method and the trans analysis incorporated in the GBAT pipeline is distinct from the two gene-based method. We did not just apply a variation of the gene-based approach to the trans regulation setting, we also very carefully designed our method such that it robustly detects high-quality trans-eQTLs with higher power and lower false positive rates.

Simply applying the gene-based (or TWAS) approach to detect trans-eQTLs will lead to severe false positive problems. For example, the Luijk et al method produced many false positive trans signals in their 2018 study for several reasons.

First, 15 out of 49 (31%) significant trans gene regulator reported by Luijk et al are likely false positives due to erroneous RNAseq reads mapping in low mappability genomic regions (Table S5). An earlier study (Saha and Battle 2019 F1000Research) have shown that reads mapped to low mappability regions could lead to up to 75% false trans signals. Indeed, 7 trans regulator genes have very low mappability scores (min=0.23, max=0.77, mean=0.57), and the remaining 8 regulators have higher mappability scores (>0.8), but they are highly cross-mappable to their trans target genes (more than one 36kmer or 75kmer can be mapped to both genes). In contrast, our method thoroughly removes problematic reads mapped to low mappability regions before quantifying gene expression, and we also carefully checked our results to further remove any cross mappable gene pairs.

Second, there is likely inflation of false positives in the remaining Luijk et al. signals. Their method used 1/3 of the samples as training data to train cis-eQTL weights, and predict the cis genetic values of gene expression in all the samples. Without at least splitting training and testing samples, the predictions are extremely prone to overfitting, and this is especially problematic in trans- signal detection. Our permutation analysis showed severe inflation of false positives (Figure 2B, Figure S1D) and our NULL simulation (trans effect = 0) also showed higher false positive rates (Figure S1C). The correct way to avoid this overfitting is through cross validation, but it will increase computational cost tremendously for whole genome trans signal scans. We solved this problem in our work.

Third, the naïve use of PEER factors or surrogate variables (SVs) in trans-eQTL association analysis is also problematic in their studies. PEER factors or SVs could capture large and wide-spread trans effects, and if they are naively included in the association model, it will lead to not only lower power, but also false positives due to collider effects (Dahl 2019 Genetics). The Luijk et al did not take this into consideration, and therefore are subject to more false positives and power loss. Our method utilized supervised versions of SVs in trans association tests to improve power and reduce false positives.

The method comparison to Luijk et al was absent in our previous manuscript. In our revised manuscript, we added comprehensive and more realistic simulations to compare our method with the Luijk et al method (and the top cis-eQTL approach, where only the top cis-eQTL of each gene is tested for trans eQTL association). We comprehensively compared the power and false positive rates of the methods (Page 6, Figure 2A, Figure S1A-C). We also performed the permutation analysis to evaluate the inflation of false positives and reported results (Page 6, Figure 2B, Figure S1D). We also emphasized and discussed the importance of considering sequence homology/mappability in trans analysis in results (Page 5) and discussion (Page 12, Table S6).

The failure to produce high-quality trans-eQTLs is not only present in the Luijk et al method, but many other trans-eQTL methods. Low power and high inflation of false positives in trans-eQTL scans is one of the main reasons for the extremely low replication rates across studies (Vosa 2018 bioRxiv). We carefully considered many aspects of power loss and false positives in our method to produce high quality trans-eQTLs. In our revised manuscript, besides demonstrating higher power in comparison to the Luijk method and SNP-based methods, we also showed that GBAT results are highly replicable both within and across studies (Results, Page 8 and 9), supporting that our method can robustly detect high quality trans-eQTLs.

In addition, our method is also unique that we do not require external training samples, and the method achieved high prediction accuracy and computational efficiency without overfitting. Our method will be very useful for studies that lack training samples that match the population background and cell type/state.

We note that we didn’t compare to Wheeler et al 2019 in our revised manuscript. The Wheeler et al 2019 manuscript was available to the public later than ours. We posted our manuscript on bioRxiv on Aug 20th 2018, and the Wheeler et al manuscript was posted three months later on Nov 19th 2018. We also submitted our manuscript to Genome Biology (March 23 2019) before they were published on Genetic Epidemiology (04 April 2019). The editorial board has agreed that it was sufficient to compare our method to the Luijk et al in the revised manuscript. We note that the efficient cross-validation approach, quality control steps, and recommended use of PEER/SVA factors are all components of this paper that are not contained in the Wheeler et al work. To inform readers about similar and available gene-based methods, we have added a sentence in the discussion (Page 12):

“Very recently, a parallel study of Wheeler et al. ((Wheeler et al. 2019), which was publicly released after our study(Liu et al. 2018)), applied gene-based approaches PrediXcan and MultiXcan to detect trans regulation signals. The method used external training samples (eg. GTEx) to obtain the weights of cis regulatory SNPs. However, we note that the use of external training samples relies on the assumption that cis effects are shared across different datasets or tissues(GTEx Consortium 2015; Liu et al. 2017). The cis effects that are tissue specific could lead to false inference of trans effects (e.g. wrong sign of effect) or loss of power (Figure S4). Our GBAT pipeline can be easily modified to incorporate gene expression prediction obtained from the prediction method in Ref(Wheeler et al. 2019). However, we suggest following the rest of the GBAT pipeline, which includes vigorous removal of problematic RNA-seq reads before quantifying gene expression, checking mappability and cross-mappability of the trans signals, and proper inclusion of supervised surrogate variables in trans association testing.”*

Reviewer #2: Main comments

1. The concept of using a cis-region based genetic predictor is interesting in that it should capture the summed genetic variance in the region. However, the strategy of reducing the test space by only fitting trans-eQTL between a significant cis-eSNP and other genes has been done routinely. One of the most powerful examples being in Westra et al. Nature Genetics 2013. Where they use the cis-eQTL top eQTL. I can see how the CVGP allows genetic effects of multiple cis-eQTL to be captured, it will be estimated with considerable great error than the top cis-eQTL. In evaluating the performance of GBAT, benchmarking over using the common cis-SNP filtering / test would be very helpful to readers.

*We thank the reviewer for the comment. In our revised manuscript, we added extra simulations to compare our method with the top cis-eQTL approach mentioned by the reviewer (Figure 2). We show that the top cis-eQTL approach is less powerful than gene-based method at different sample sizes and causal proportions (0.5%,1% and 5%). Our simulation results are in agreement with previous findings in the TWAS paper (Gusev 2016 Nat Genet, Figure 5), where they also showed that their gene-based method (TWAS) is generally much more powerful than the top cis-eQTL approach. The only exception when the top cis-eQTL approach had more power is when genotypes of all SNPs are known and there is only one cis-eQTL causal to the gene, which is not realistic(GTEx Consortium 2015).*


2. In results page 2 lines 45-48 it is noted that including conditional CV as covariates is impossible in SNP based trans-associations. Firstly, it is not clear why this this impossible. Secondly, the common was to include SV is to use them to first correct the expression levels of gene i, then use the corrected values in the eQTL analysis. Some additional clarification and justification of these statements would be beneficial. Regressing normalised Ej on SVi has also been done by others - for example see Lloyd-Jones et al. AJHG 2017.

*We thank the reviewer for pointing this out. We have revised the sentence (Page 5) to :” We note that including conditional SVs as covariate is much more computationally efficient in our gene-based approach than in traditional SNP based methods for genome-wide scans of trans-eQTLs.”

We agree with the reviewer that a common practice is to first use SV to correct the gene expression levels, and then use the residuals for association testing, eg in Lloyd-Jones et al. AJHG 2017. However, as explained in several previous work(Joo et al. 2014; Kang et al. 2008; Dahl et al. 2019), SVi, which is the conditional SV, will further improve power and reduce false trans positives, and SVi was not used in the Lloyd-Jones et al.*


3. Demonstration of the performance of GBAT needs to show that replication is higher compared to other methods. Rather than being 'out of scope' I believe this is would provide a critical piece of information for the manuscript and in convincing researchers to adopt the method.

*We thank the reviewer for the comments and we agree that performance comparison to other methods is a critical piece of information that would greatly improves the manuscript. Therefore, we did the following in the revised manuscript: (1) power comparison of GABT with two SNP-based methods and one gene-based methods (Luijk et al 2018 Nat Comm) in simulations; (2) false positive rates comparison and evaluating inflation of false positives through permutation analyses in simulations. (3) found extensive false positive trans signals due to read mapping issues in the results reported by Luijk et al. (4) demonstrated high replication rates of GBAT in read data analysis.

More specifically, we first showed that GBAT is more powerful than the two SNP-based methods (traditional SNP-based trans-eQTL scan and the top cis-eQTL approach mentioned by the reviewer) at all simulated sample sizes and genetic architectures (Figure 2A). GBAT is more powerful than the Luijk method at lower sample sizes (N<=600). At higher sample sizes, the power of GBAT is comparable to the Luijk method (Figure 2A). However, we found Luijk et al has higher false positive rates at higher sample sizes in our null simulations (trans effect=0, Figure S1B). In addition, through permutation analysis, we found the Luijk method has a severe inflation of false positives (Figure 2B, Figure S1D).

We also found that the Luijk et al method produced many false positive trans signals in their 2018 study. First, 15 out of 49 (31%) significant trans gene regulator reported by Luijk et al are likely false positives due to erroneous RNAseq reads mapping in homologous/low mappability genomic regions (Table S5). An earlier study (Saha and Battle 2019 F1000Research) have shown that reads mapped to low mappability regions could lead to up to 75% false trans signals. Indeed, 7 trans regulator genes have very low mappability scores (min=0.23, max=0.77, mean=0.57), and the remaining 8 regulators have higher mappability scores (>0.8), but they are cross mappable to their trans target genes (Table S5, more than one 36kmer or 75kmer can be mapped to both genes). Our method thoroughly removes problematic reads mapped to low mappability regions before quantifying gene expression, and we also double checked our results to further remove any cross mappable gene pairs. Second, we believe there are more false positive trans signals remaining in their work, as evidenced by the inflation of false positives in permutation and simulation analyses (Figure 2 and Figure S1).

In our revised manuscript, we also reported high replication rates of GBAT both within and across studies (Page 8 and Page 9). First, 92% of the GBAT trans gene pairs were replicated in SNP-based scans of trans-eQTL using Matrix eQTL. Second, by splitting DGN dataset into two non-overlapping subsets, 87% (resp. 73%) of trans signals were replicated in the other subset. Third, we also looked at the replication of our significant GBAT trans signals (at 10% FDR) in DGN versus the significant trans-eQTL results reported by eQTLGen (at 5% FDR), which is the largest whole blood RNA-seq dataset. We found 111 of 166 (67%) GBAT signal were also significant in eQTLGen at 5% FDR, which demonstrated high replication rate of significant trans-eQTLs across studies and supported that GBAT can robustly detect high quality trans signals.*

4. Filtering multi-mapped reads is of course important. However, this is very standard in most RNA seq studies. For example, it is default in HTseq, eQTL-cookbook, and the GTEx pipelines. I think the paper would benefit from not positioning this as a key step. It detracts from the novelty, and also leads to the impression that most other analysis pipelines/tools do not follow this approach, which is not true.

*We agree with the reviewer that several pipelines and analysis removed multi-mapped reads. Therefore, in the revised manuscript, we deleted “removing multi-mapped reads” as a key step of the method (Page 4 and Figure 1).
Read mapping issues is a major cause of false positives for trans analysis. To reduce false positives, we note that in addition to multi-mapped reads, we also thoroughly removed RNA-seq reads that are mapped to low mappability regions (homologous regions) of the genome, even though these reads are marked as “uniquely mapped” by the read mapping algorithms. In addition, we thoroughly checked cross mappability between the regulator genes and their trans targets to further remove false positive signals. We emphasized these steps in the GBAT method overview on Page 5.

Even though removing multi-mapped reads has been used in the pipelines mentioned by the reviewer, the importance of checking gene mappability and cross mappability is still under-valued in many trans studies. For example, the Luijk 2018 study did not mention if they removed any multi-mapped reads or reads mapped to low mappability regions. Indeed, many of their trans signals are likely false signal due to read mapping issues. We therefore added extra discussion about the effects of reads mapping and sequence similarity on detecting trans associations on Page 12.*

5. The inflation of the false positive rate using the regression model E_j = CVGP_i + SV_i is intriguing. It is not clear why this is happening given that correcting E_j by SV_i then fitting E_j = CVGP_i is ok. The paper would benefit considerably if an explanation for the inflation can be demonstrated.

*We thank the reviewer for the comment. Through careful examination, we found that the inflation of false positives can happen when the covariates (such as SVs) are very heavy tail distributed, because regressing on heavy tailed covariates results in heavy tailed residuals. However, we think it is not common for covariates to be extremely heavy tailed. Therefore, we removed discussion and emphasis on the false positive inflation caused by the regression model E_j = CVGP_i + SV_i in the revised manuscript. Nonetheless, we still used quantile normalized residuals in our regression model, as this guarantees the normal distribution of the residuals.*

6. The need to reduce the number of tests is important, but the approach of only testing cis-eQTL means that all 'trans-trans' eQTL will be missed. This is worth some discussion as it is anticipated that the majority of genetic variation for gene expression is acting trans-trans, although with smaller effects than cis-trans. The 1 sentence in the discussion just mentioned this as a limitation.

*We thank the reviewer for the comment. In the revised manuscript, we added the following discussion (Page 12): “Second, trans regulatory effects that do not function through cis expression regulation will not be detected by GBAT. The effects of trans-eQTLs might be mediated by other cis-regulatory mechanisms such as splicing and methylation, or even mediated by other trans mechanisms. Further improvement of GBAT that incorporate these effects will further improve power of trans- detection.”*

7. Simulating 10% of SNPs being causal is extremely high and unrealistic. Some justification for these percentages needs to be given based on what is known about eQTLs.

*We agree with the reviewer that simulating 10% of SNP being causal is high. Thus, we simulated the causal proportions at 0.5%, 1% and 5% (Page 6 and Figure 2). We note that the power of GBAT is comparable at different causal proportions.*

8. It is unclear if the power of GBAT was tested at the same alpha level to SNP-based methods, or at a test-specific multiple testing corrected thresholds. It should be compared at the same alpha.

*We thank the reviewer for pointing this out. In the revised manuscript, we used Benjamini-Hochberg procedure and compared power at 5% FDR for all method (Page 6 and Figure 2).*

9. The results reporting the GO enrichment are underwhelming, and it is not clear what conclusions are drawn from these. Statements such as 'the results are encouraging as these categories are expected to have broad trans effects' really should be supported by references or qualified.

*We apologize for being unclear about the results on GO enrichment. In the revised manuscript, we have reduced the emphasis on the GO results by reducing the amount of text describing this analysis, and we also changed the text (Page 9) to:” The results support a well-known trans regulatory mechanism that many regulators encode gene products, such as transcription factors, that bind to distant DNA sequences and regulate the expression of the target genes in trans.”.*


Minor comments

1. Slightly pedantic, but using the word 'suffer' (line 22) when describing multiple testing correct can give the impression that multiple testing is a bad thing. Could this be re-phrased to explain that the issue is one of power? Irrespective of effect sizes, the nominal alpha level that needs to be reached is smaller, thus given the same effect size large sample sizes are needed.

*We thank the reviewer for the comment and we changed the text containing “suffer” to a neutral word “have”.*

2. The comment that cis-eQTL are largely shared across tissues is not really true. The GTEX papers showed that some cis-EQTL are truly tissue ubiquitous, many are shared by only a handful of tissue types, and some tissue specific.

*We agree with the reviewer that tissue specific cis-eQTLs exist. We removed the mention of the largely shared cis-effects from discussion in the revised manuscript, and the text (Page 11) now reads:
“we note that the use of external training samples relies on the assumption that cis effects are shared across different datasets or tissues”.*

3. Are some of the methods are missing? The methods section starts with 'Second, GBAT uses…. 'It's possible this is a typo, as most of the key methods descriptions are included.'

*We thank the reviewer for pointing this out. It was a typo and has been corrected in the revised version.
 
Bibliography

Dahl, A., Guillemot, V., Mefford, J., Aschard, H. and Zaitlen, N. 2019. Adjusting for principal components of molecular phenotypes induces replicating false positives. Genetics 211(4), pp. 1179–1189.
GTEx Consortium 2015. Human genomics. The Genotype-Tissue Expression (GTEx) pilot analysis: multitissue gene regulation in humans. Science 348(6235), pp. 648–660.
Joo, J.W.J., Sul, J.H., Han, B., Ye, C. and Eskin, E. 2014. Effectively identifying regulatory hotspots while capturing expression heterogeneity in gene expression studies. Genome Biology 15(4), p. r61.
Kang, H.M., Ye, C. and Eskin, E. 2008. Accurate discovery of expression quantitative trait loci under confounding from spurious and genuine regulatory hotspots. Genetics 180(4), pp. 1909–1925.
Liu, X., Finucane, H.K., Gusev, A., et al. 2017. Functional architectures of local and distal regulation of gene expression in multiple human tissues. American Journal of Human Genetics 100(4), pp. 605–616.
Liu, X., Mefford, J.A., Dahl, A., et al. 2018. GBAT: a gene-based association method for robust trans-gene regulation detection. BioRxiv.
Wheeler, H.E., Ploch, S., Barbeira, A.N., et al. 2019. Imputed gene associations identify replicable trans-acting genes enriched in transcription pathways and complex traits. Genetic Epidemiology 43(6), pp. 596–608.*

**Second round of review**

**Reviewer 1**

I am grateful the authors have now compared their method to other methods. The authors highlight that GBAT properly accounts for multi mapping reads, which is indeed a highly valuable procedure, although this strategy has been described before (Saha and Battle, F1000Research 2018).

**Reviewer 2**

The authors have addressed all go my comments satisfactorily
